# Melon13-lipoxygenase *CmLOX18* may be involved in C6 volatiles biosynthesis in fruit

**DOI:** 10.1038/s41598-017-02559-6

**Published:** 2017-06-06

**Authors:** Chong Zhang, Songxiao Cao, Yazhong Jin, Lijun Ju, Qiang Chen, Qiaojuan Xing, Hongyan Qi

**Affiliations:** 1 Key Laboratory of Protected Horticulture of Education Ministry and Liaoning Province, College of Horticulture, Shenyang Agricultural University, Collaborative Innovation Center of Protected Vegetable Suround Bohai Gulf region, Shenyang, Liaoning 110866 P.R. China; 2 College of Horticulture, Jilin Agricultural University, Changchun, P.R. China; 3 College of Agriculture, Heilongjiang Bayi Agricultural University, Daqing, Heilong jiang163319 P.R. China; 4 Experimental Center, Shenyang Normal University, Shenyang, Liaoning 110034 P.R. China

## Abstract

To better understand the function role of the melon *CmLOX18* gene in the biosynthesis of C6 volatiles during fruit ripening, we biochemically characterized *CmLOX18* and identified its subcellular localization in transgenic tomato plants. Heterologous expression in yeast cells showed that the molecular weight of the *CmLOX18* protein was identical to that predicted, and that this enzyme possesseed lipoxygenase activity. Linoleic acid was demonstrated to be the preferred substrate for the purified recombinant *CmLOX18* protein, which exhibited optimal catalytic activity at pH 4.5 and 30 °C. Chromatogram analysis of the reaction product indicated that the *CmLOX18* protein exhibited positional specificity, as evidenced by its release of only a C-13 oxidized product. Subcellular localization analysis by transient expression in *Arabidopsis* protoplasts showed that *CmLOX18* was localized to non-chloroplast organelles. When the *CmLOX18* gene was transgenically expressed in tomato via *Agrobacterium tumefaciens*-mediated transformation, it was shown to enhance expression levels of the tomato hydroperoxide lyase gene *LeHPL*, whereas the expression levels of six *TomLox* genes were little changed. Furthermore, transgenic tomato fruits exhibited increases in the content of the C6 volatiles, namely hexanal, (Z)-3-hexanal, and (Z)-3-hexen-1-ol, indicating that *CmLOX18* probably plays an important role in the synthesis of C6 compounds in fruits.

## Introduction

Aroma volatiles are vital characteristic that determine the quality and commercial value of fruits. Different fruits have different suites of volatile compounds, which contribute to their unique aromas^1^. For example, in apple, strawberry, and melon, esters are the main flavour compounds that impart a fruity flavour^2–4^. Previous studies have indicated that degradation of linoleic and linolenic acids contribute to the synthesis of esters, containing 2, 4, and 6 carbon straight chains^5, 6^. Lipoxygenase (LOX) enzymes might contribute to the breakdown of long-chain fatty acids to C6 aldehydes by hydroperoxide lyases (HPL), which were further converted to alcohols by aldehyde dehydrogenase (ADH), followed by the conversion of alcohols to esters by alcohol acetyl transferase (AAT)^7–9^.

Plant LOX enzymes, which are encoded by a multi-gene family, are 95–100 kDa monomeric proteins containing an N-terminal β-barrel domain (25–30 kDa) that is probably involved in membrane or protein interactions, and a C-terminal α-helix-rich domain (55–65 kDa) containing the catalytic site^10^. On the basis of their positional specificity, LOX enzymes can be classified into three types: 9-LOXs, 13-LOXs, and 9/13-LOXs^11^. 9-LOX and 13-LOX enzymes can produce 9- and 13-hydroperoxy products, respectively, whereas 9/13-LOXs can produce both 9- and 13-hydroperoxy products^12, 13^. Heterologous expression of plant LOXs in *Escherichia coli* or yeast has enabled elucidation of the positional specificity for substrate oxygenation. A few of the 9/13-LOXs, such as maize *ZmLOX1* ^14^, pea *PsLOXN2* ^15^, rice *OsLOX1* ^16^, and tea *CsLOX1* ^17^, have been shown to confer dual positional specificity since they release both C-9 and C-13 oxidized products in equal proportions.

Different LOX isoforms initiated the synthesis of functionally diverse products, and thus LOXs have been regarded to play different roles in plant development and ripening^18^. 9-LOXs were implicated in diverse physiological processes in plants, such as growth and development -^19, 20^, defence^21, 22^, and resistance to pathogens^23^. In contrast, 13-LOXs have been reported to produce the ‘green note’ compounds in plant tissues^24^, and also played an important role in wound- and herbivore-induced jasmonate (JA) accumulation^25–27^. Furthermore, the subcellular location of a protein is very important because it is closely related to that proteins biological function^28^. LOX enzymes have been found in various organelles of plants, which could determine the specific function of the different LOXs^29^. For example, tomato 13-lipoxygenase TomloxC was localized in the chloroplast, where it was specifically involved in the generation of fatty acid-derived straight-chain five- (C5) and six- (C6) carbon flavour compounds (aldehydes and alcohols) in both fruit and leaves^30, 31^. In potato, LOX H1 and LOX H3 were localized in both the chloroplast stroma and thylakoids, and both of these enzymes were involved in the synthesis of JA and C-6 aldehydes^32, 33^. Other LOX proteins, for example, cucumber LOX, were found in microsomal membranes^34^, whereas soybean LOX was found in both the cytosol and vacuole^35, 36^.

In oriental melon, the genes *CmLOX01*, *CmLOX03*, and *CmLOX18* were expressed during fruit ripening^37^. The 13-LOXs *CmLOX03* and *CmLOX18* were potentially important candidate genes involved in straight-chain ester production in melon; however, further studies are needed to verify this supposition^38^. In the present study, we transformed the 13-lipoxygenase *CmLOX18* gene into tomato and analysed its enzymatic characteristics by using the recombinant protein expressed in yeast. Our results showed that *CmLOX18* was a member of the 13-LOX family and was localized in non-chloroplast organelles. Transgenic tomato plants over-expressing *CmLOX18* exhibited a significant increase in the biosynthesis of C6 compounds; however, the biosynthesis of C5 compounds were not changed. In addition, the expression levels of members of the *Tomlox* gene family were not altered in the transgenic plants. Therefore, we concluded that a critical role for *CmLOX18* was the formation of C6 compounds, such as hexanal, (Z)-3-hexenal, and (Z)-3-hexenol, in fruit, and that it might be involved in straight-chain ester production in melon.

## Results

### Biochemical activity and characterization of recombinant yeast *CmLOX18*

**Figure 1:**
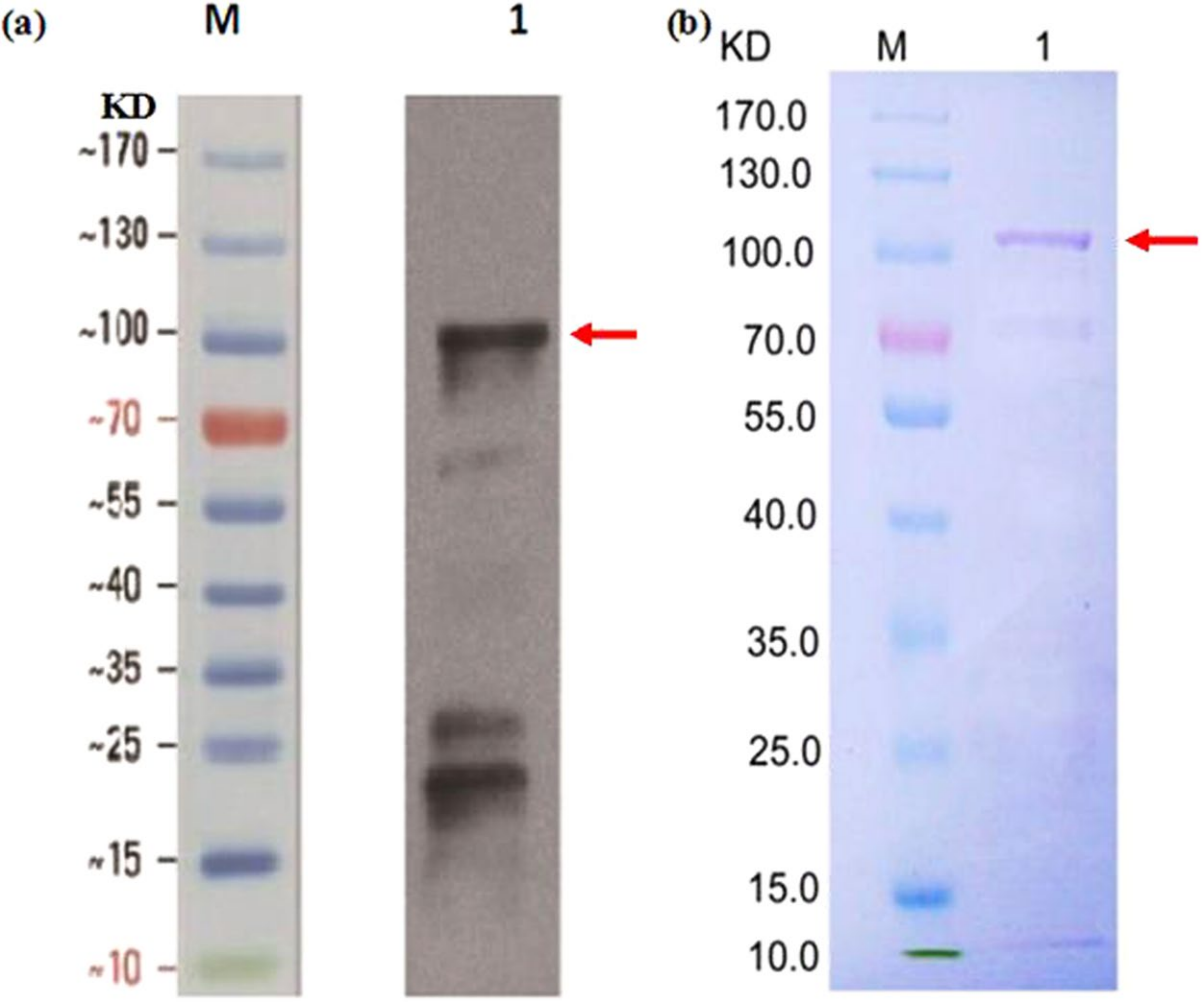
Analysis of recombinant *CmLOX18* (**a**) Western blot analysis. Total proteins (20 μg) were isolated from yeast cells expressing *CmLXO18* which was harvested at 24 hours induction and separated on 12% SDS/PAGE gel, then transferred to a PVD membrane and LOX detected with anti-His antibody. (**b**) SDS-PAGE analysis of the recombinant *CmLOX18*. The proteins were stained with Coomassie brilliant blue R-250. M: Positions of marker protein. 1: SDS-PAGE of the purified CmLOX18 extracted from pYES2/lox transformed yeast cultures (20 μg). The expressed *CmLOX18* was indicated by red arrows.

The ORF of *CmLOX18* was cloned into a vector pYES2.1 for expression in yeast to characterize the enzymatic activity of the encoded protein. The yeast containing the recombinant plasmid grew in selective medium (SC-U) with 2% galactose as inducer of the recombinant protein expression. The recombinant lipoxygenase encoded by *CmLOX18* was expressed and purified. Heterologously expressed *CmLOX18* was identified by western blot analysis using an anti-His-tag antibody. Western blotting and SDS–PAGE analysis revealed one unique band of approximately 100 kDa protein and were in good agreement with the predicted molecular mass of *CmLOX18* (approximately 102.1 kDa) (Fig. 1).

**Figure 2:**
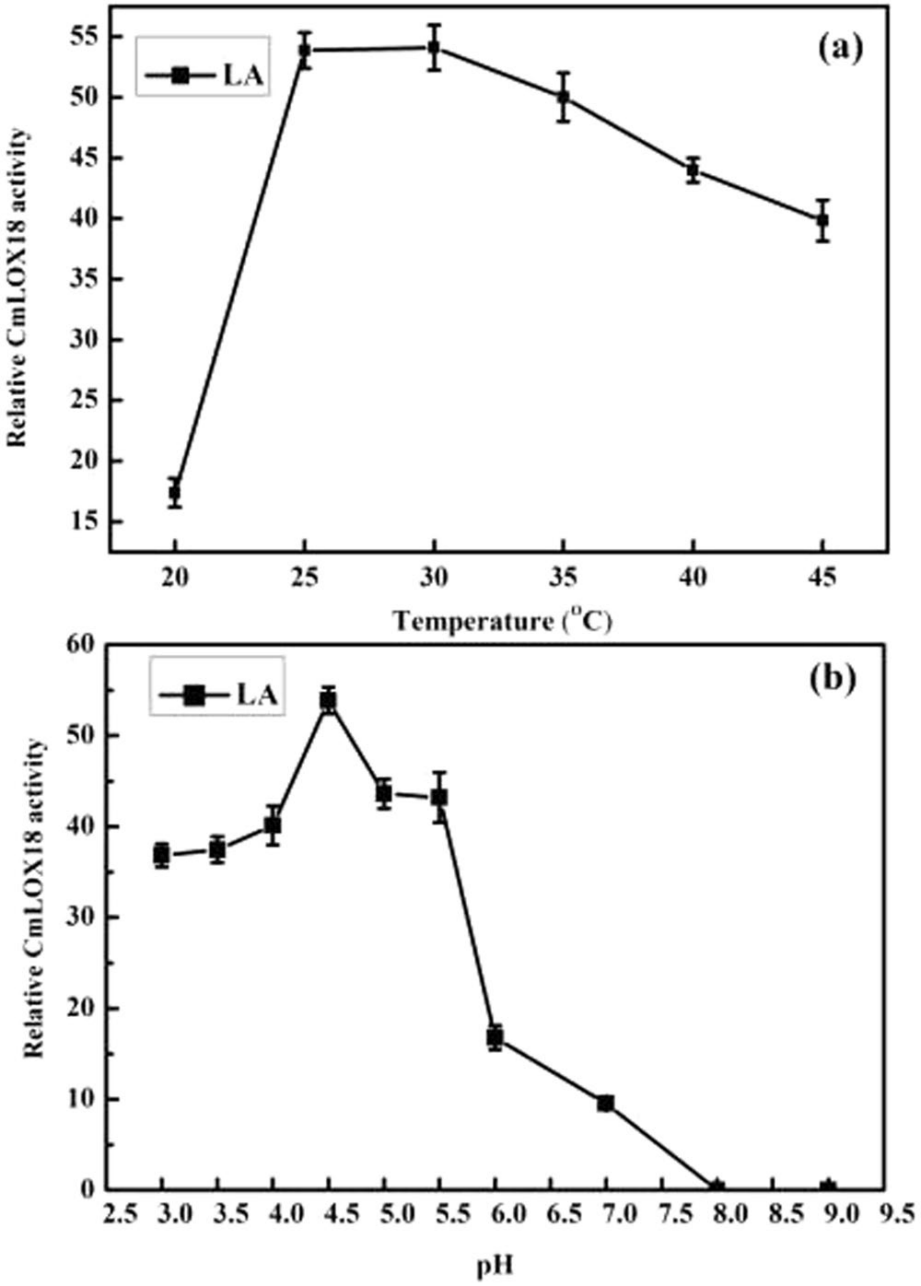
The optimum temperature (**a**) and Ph (**b**) for recombinant CmLOX18. The effects of pH and temperature on the enzymatic activity of recombinant *CmLOX18* were determined using linoleic acid (LA) as substrates. The recombinant *CmLOX18* proteins displayed the highest catalytic activity at pH 4.5 (**a**) respectively. The optimum temperatures for *CmLOX18* were observed at 30 °C (**b**), respectively. The maximum activity was estimated as 100%. Means ± SD were obtained from three independent measurements.

Biochemical activity of the purified recombinant *CmLOX18* was investigated by measuring the increase in A_234_ using linoleic acid as substrates. To optimum pH was determined by varying in the pH values of the reaction buffers from 3.0 to 9.0. At 25 °C, the optimum pH turned out to be 4.5 since *CmLOX18* showed the highest activity at that pH. By contrast, *CmLOX18* activity decreased by 32% at pH 3.0 and by 82% at pH 7.0, respectively, and was almost completely deactivated at pH 8.0–9.0 (Fig. 2a). To determine the optimum temperature for recombinant *CmLOX18*, enzyme activity was measured over a range of temperatures (20 °C–45 °C). Maximal activities for recombinant protein were observed at 30 °C, while the activity of the protein exhibited the lowest activity at 20 °C (Fig. 2b).

The kinetic parameters were analysed using linoleic and linolenic acids as substrates (Fig. S2). The recombinant *CmLOX18* showed 3.12-fold higher *K*_m_ value for linoleic acid (126.9 μM) than for linolenic acid (40.72 μM). Comparison of the *V*_max_ values showed that *CmLOX18* oxidized linoleic acid approximately 4-fold faster than linolenic acid. The *k*_cat_/*K*_m_ values of recombinant *CmLOX18* for linoleic acid and linolenic acid were 34.54 and 26.96 s^−1^ μM^−1^, respectively (Table 1). These results indicated that linoleic acid was clearly the preferred substrate for the recombinant *CmLOX18*.

**Table 1:** Kinetic parameters of purified recombinant CmLOX18.

Substrate	Km (μM)	Vmax (μmol s^−1^)	Kcat (s^−1^)	Kcat/Km (s^−1^ μM^−1^)
Linoleic acid	126.9 ± 18.6	32.87 ± 3.1	4383 ± 295	34.54 ± 2.7
Linolenic acid	40.72 ± 2.65	8.19 ± 0.8	1098 ± 78	26.96 ± 2.2

Kinetic parameters were calculated using a Michaelis-Menten plot analysis program. Values represent the mean ± SD of 3 independent replicates. Kcat is defined to equal Vma/Et. Et is total enzyme concentration is molar.

**Figure 3:**
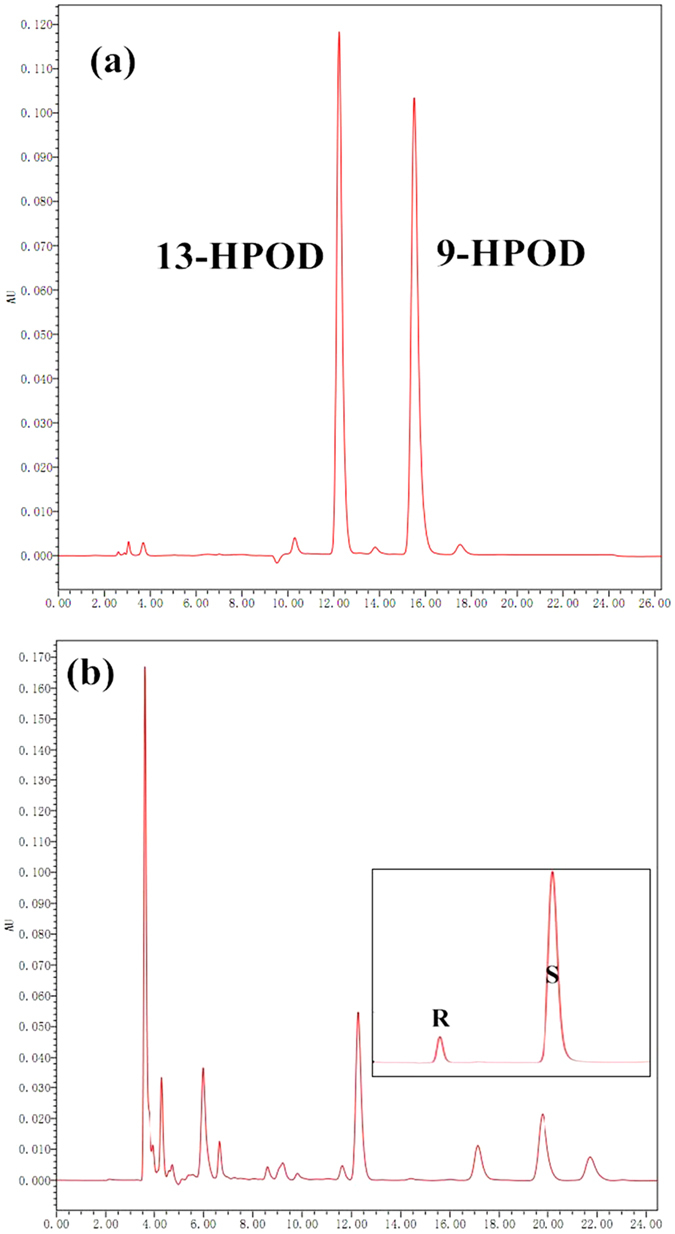
Determination of positional specificity of the recombinant *CmLOX18*. (**a**) The retention time of the isomers produced by soybean LOX1 compared with authentic standards of 13-HPOD and 9-HPOD. (**b**) SP-HPLC analysis of the reaction mixture catalysed by recombinant *CmLOX18*. Boxes: chiral-phase HPLC showing the enantiomer composition of 13-HPOD.

In order to study the positional-specificity of recombinant *CmLOX18*, we separated the reaction products by SP-HPLC. The retention time of *CmLOX18* product was consistent with authentic standards of 13-HPOD (Fig. 3a), which indicated that *CmLOX18* was 13-LOX. The stereochemistry of the reaction products was analysed chiral-phase HPLC.13-HPOD was predominantly in the S configuration, indicating that it had been derived from the activity of a specific enzyme (Fig. 3b). The result was consistent with the positional specificity rule of plant LOXs, in which S/CF motif at the active site indicated the LOXs were 13-LOXs.

### Subcellular localization of *CmLOX18* protein

**Figure 4:**
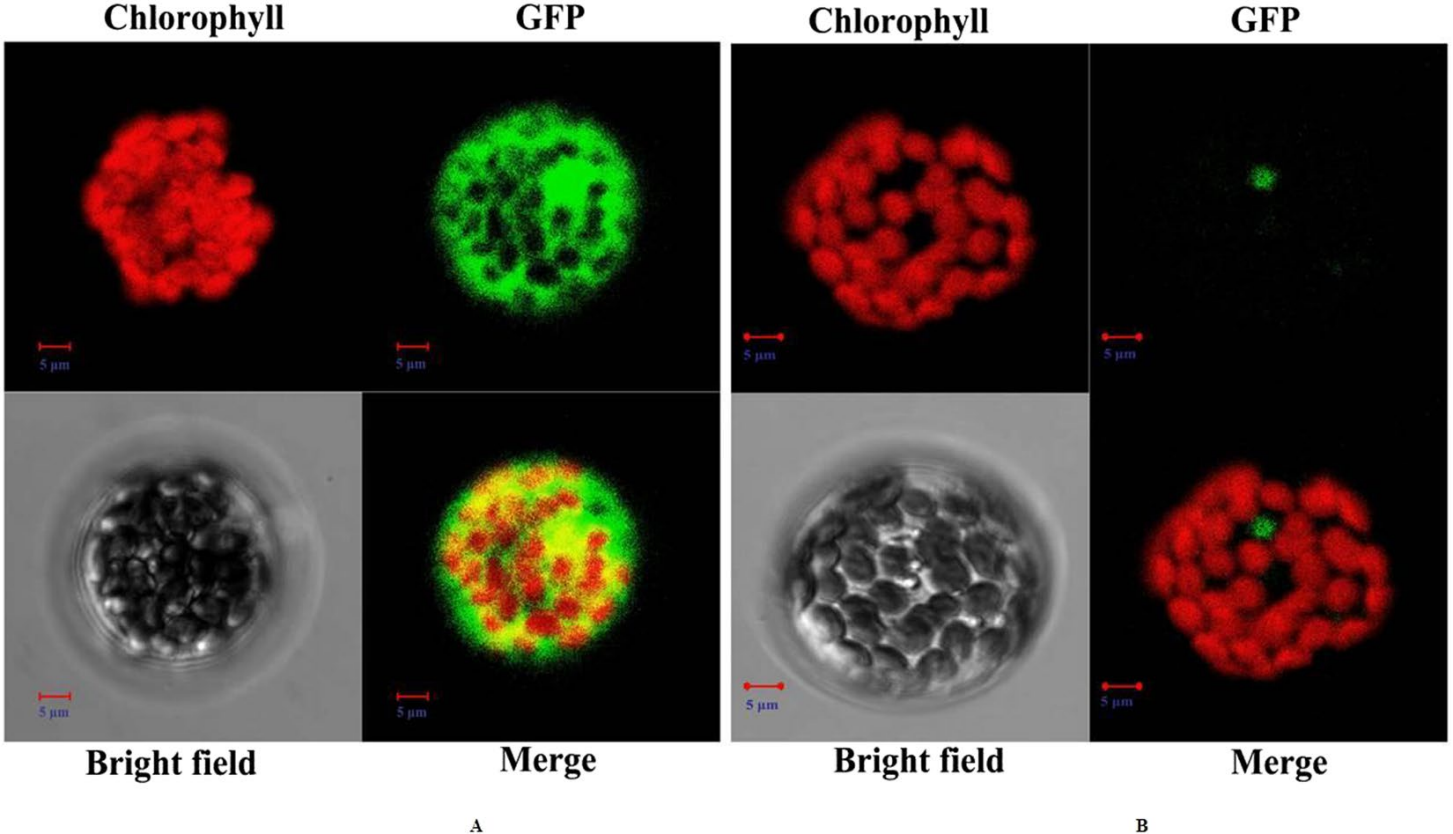
Subcellular localization of oriental melon *CmLOX18* in *Arabidopsis* protoplasts. *Arabidopsis* protoplasts were transformed by the vectors containing the 35S-GFP (**A**) and 35S-*CmLOX18*-GFP (**B**), respectively. Chlorophyll and GFP fluorescence were examined using Zeiss LSM510 confocal microscope excited with a 488-nm Ar laser line. Merge is the computed overlay of the two fluorescence images. Reference bar is 5 μm.

To confirm the subcellular location of *CmLOX18*, a green fluorescent protein (GFP) reporter gene was fused in-frame to the N-terminus of *CmLOX18* and transiently transformed into *Arabidopsis* protoplasts under the control of the 35 S promoter. As shown in Fig. 4, *CmLOX18* was not observed in chloroplasts, which was not in accord with the result of the bioinformatics analysis, and the results showed that *CmLOX18* was localized in non-chloroplast organelles.

### The identification of transgenic tomato plants over-expressing *CmLOX18*

**Figure 5:**
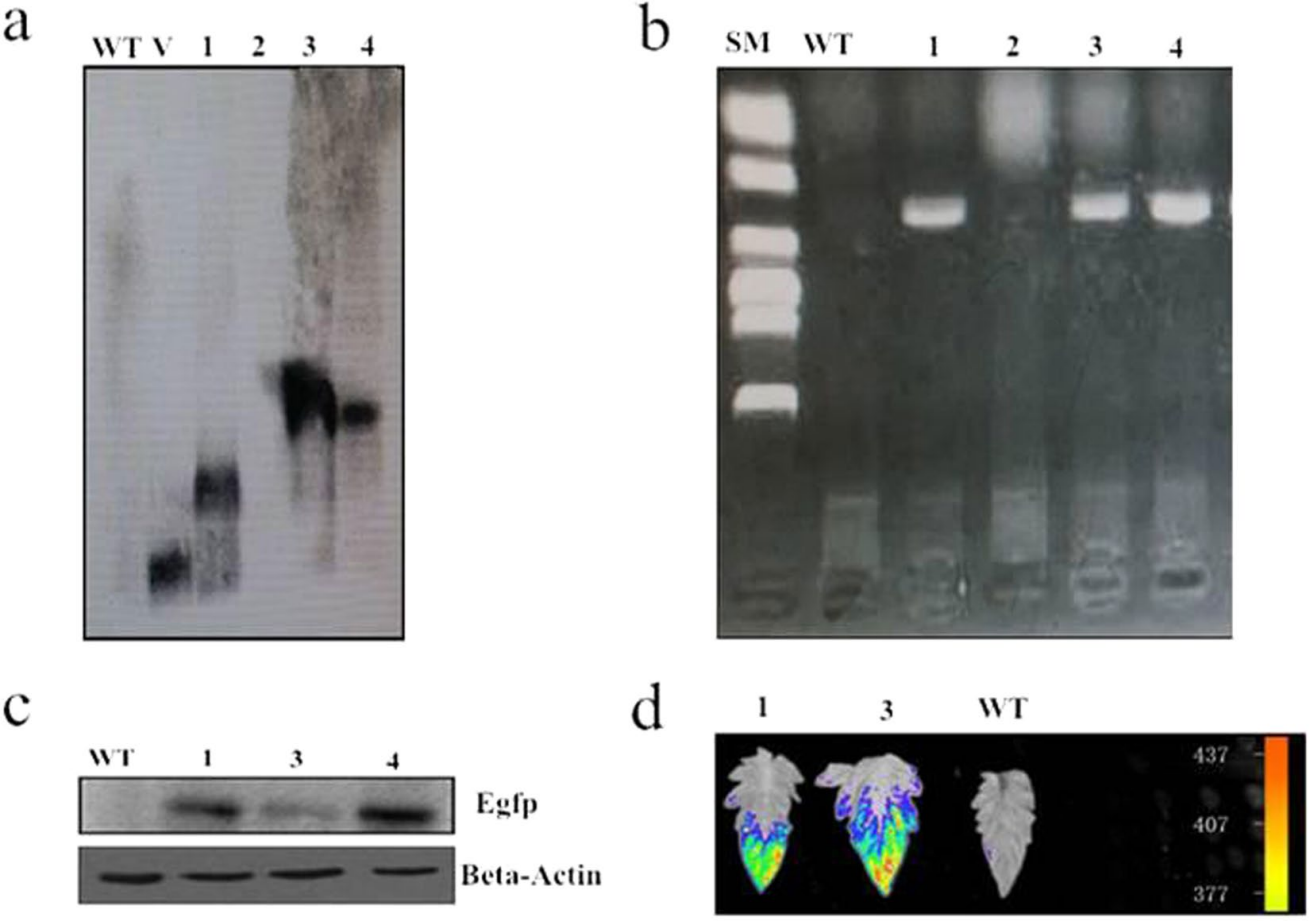
Molecular analysis of T0 transegenic tomato liners expressing *CmLOX18*. (**a**) Southern blot of wild-type control and four T0 transgenic tomato lines expression *CmLOX18*. Genomic DNA was prepared from young leaf material from wild-type control and transgenic plants: 1, 2, and 3 and V (35S-overexpression *CmLOX18* construct). The genomic DNA (10 μg/lane) was digested with HindШ and separated in a 0.8% (w/v) agarose gel. Blotted DNA was hybridized to a probe prepared from the bar gene. (**b**) Detection of *CmLOX18* cDNA by PCR analysis. Genomic DNA was extracted from transgenic lines. (**c**) Western blot. Proteins were extracted and Western blot was carried according to standard procedures using anti-e GFP Mouse Monoclonal antibody. (**d**) Images of wild-type control and twoT0 transgenic tomato leaves.

To examine the physiological role of *CmLOX18*, the full-length cDNA of *CmLOX18* was cloned into pB7FWG2.0 under the control of the 35 S promoter (Fig. S1) and transformed into ‘Zhongshu 6’ using the *Agrobacterium* method. The construct, encoding a carboxy-terminal fusion of *CmLOX18* onto EGFP under the control of a cauliflower mosaic virus 35 S promoter, contained bialaphos acetyltransferase gene (Bar) and enhanced green fluorescent protein gene (Egfp). Southern blot, PCR, Western blot, and fluorescence imaging technique indicated that three T_0_ transgenic plants were obtained (Fig. 5). Three independent transgenic lines (CM18-01, 03, and 04) which contained one copy identified by Southern blot were selected.

In comparison with “Zhongshu 6” tomato, as control plant, which have no expected bands by Southern and PCR, transgenic tomato plants indeed contained the *CmLOX18* gene (Fig. 5a and b). Furthermore, western blot (Fig. 5c) and image (Fig. 5d) analysis indicated that Egfp protein was high expressed in lines (CM18-01, 03, and 04). These results indicated that the *CmLOX18* gene was successfully transferred into tomato plant.

### Real-time qPCR analysis of LOX gene from ripening fruit of wild-type and transgenic plants

**Figure 6:**
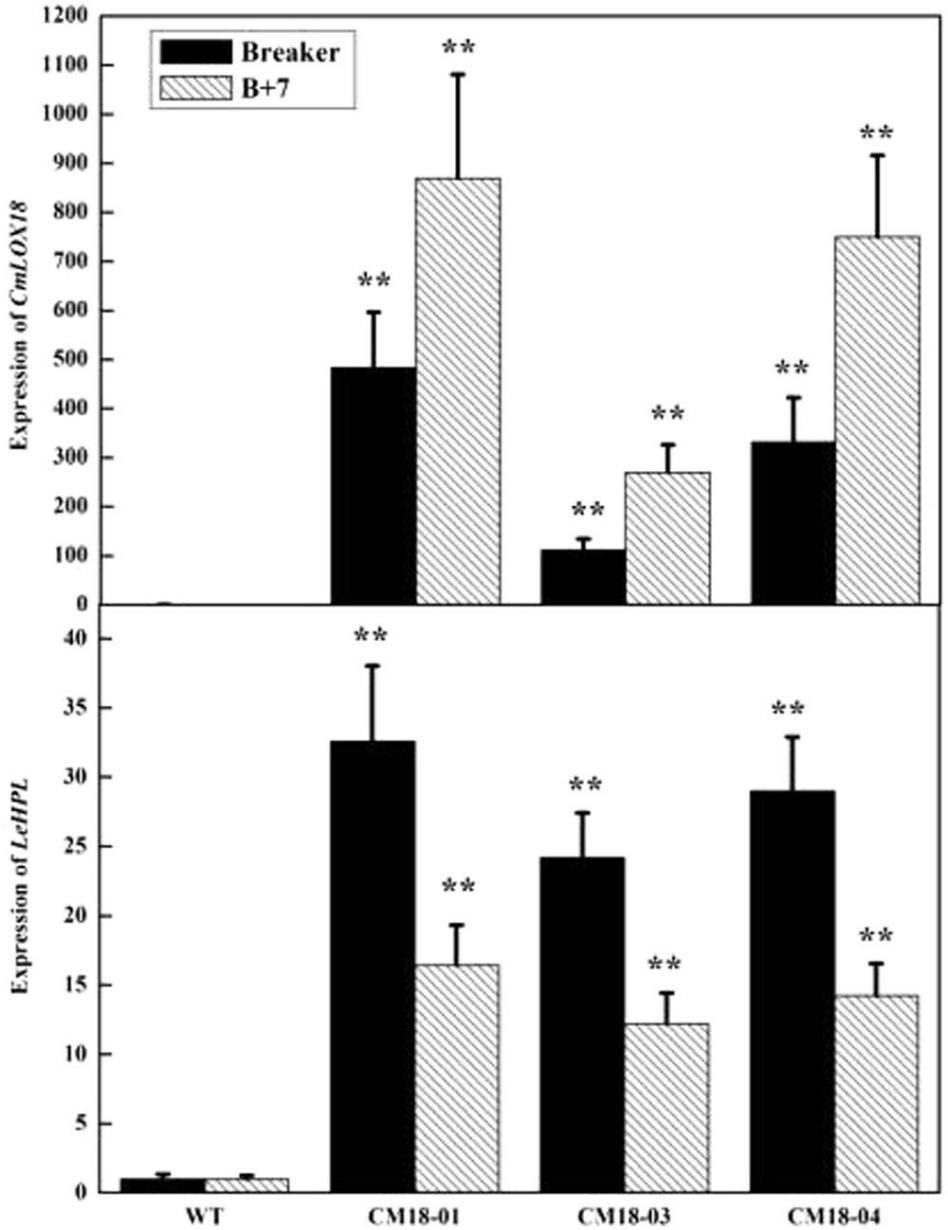
Transcript levels of *CmLOX18* and *LeHPL* in wild type (“Zhongshu 6”) and transgenic (CM18-01, 03 and 04) ripe fruits. Transcripts were quantified by Quantitative PCR. Values are mean ± standard error of three replicates. Significant differences are indicated with asterisks above the bars: *P ≤ 0.05, **P ≤ 0.01.

Real-time PCR analyses indicated that *CmLOX18* transcription were only present in three lines of *35 S::CmLOX18* (CM18-01,03 and 04), which was not detected in the wild-type. The expression level of *CmLOX18* mRNA in the fruits of three transgenic lines at the B + 7 stage were about 2-fold higher than that at the breaker stage (Fig. 6a). To find out whether the *CmLOX18* gene was involved in catalyzing chain cleavage of hydroperoxides to release C5 and C6 volatiles, real-time quantitative RT-PCR was carried out. Transcript analysis of *LeHPL* was significantly higher in transgenic fruits than that in wild-type fruits. However, the expression level of *LeHPL* at the B + 7 stage was lower than that at the breaker in both transgenic and wild-type fruits, which was not consistent with the expression of *CmLOX18* (Fig. 6b).

**Figure 7:**
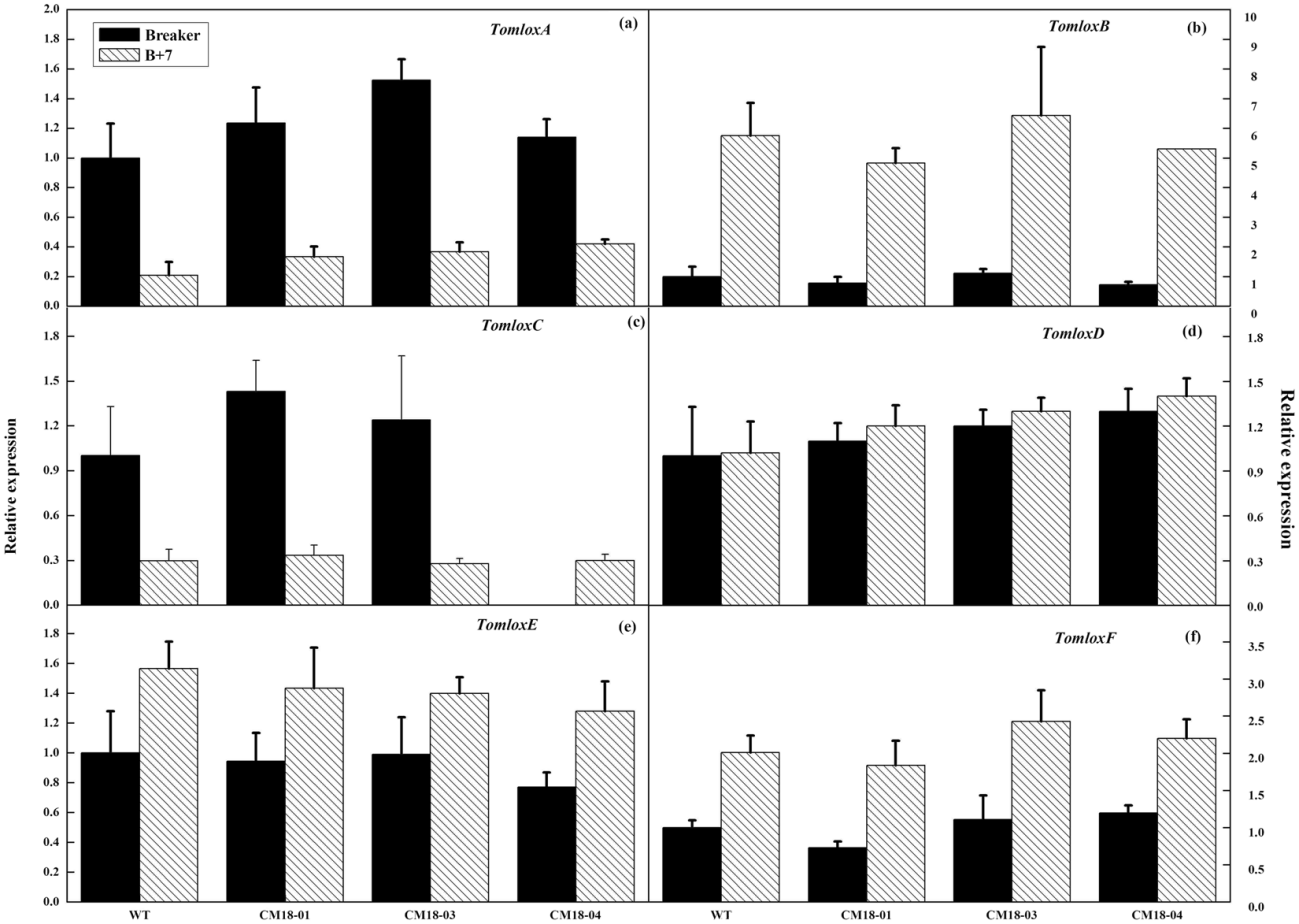
Transcript levels of *TomLOXA-F* in wild type (“Zhongshu 6”) and transgenic (CM18-01, 03 and 04) ripe fruits. Transcripts were quantified by Quantitative PCR. Values are mean ± standard error of three replicates. Significant differences are indicated with asterisks above the bars: *P ≤ 0.05, **P ≤ 0.01.

In order to confirm whether the over-expression of *CmLOX18* gene caused significant changes in the mRNA expression levels of six tomato *LOXs* in the transgenic fruits, we analyzed the expression levels of *TomloxA-TomloxF* in transgenic and wild-type fruits. The results showed that there was no difference between the expression levels of *TomloxA*, *TomloxB*, *TomloxC*, *TomloxD*, *TomloxE*, and *TomloxF* mRNA in the transgenic and wild-type fruits at the breaker and B + 7. Furthermore, the transcription levels of *TomloxA* and *TomloxC* were consistent with the *LeHPL* in both transgenic and wild-type fruits and higher at the breaker than that at the B + 7. In contrast, transcription levels of *TomloxB, TomloxE*, and *TomloxF* were lower at the breaker than at the B + 7, while *TomloxD* had no difference (Fig. 7). Taken together, the results indicated that the over-expression of *CmLOX18* caused significant changes in the expression levels of *LeHPL*, but there was no change in *Tomloxs*.

### C5 and C6 volatiles from the transgenic and wild-type fruits

**Figure 8:**
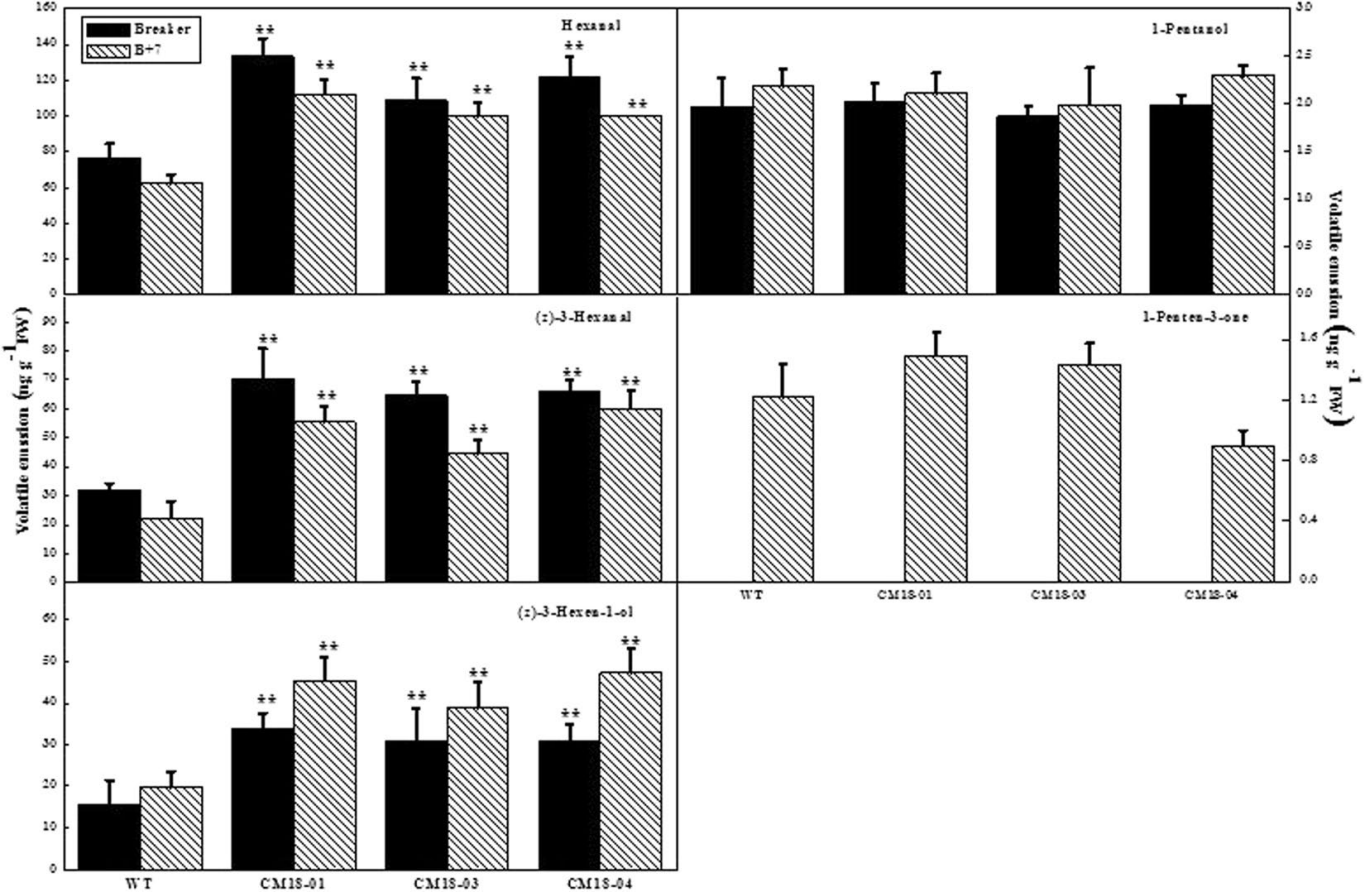
Overexpression *CmLOX18* in fruits C6 and C5 volatiles emission in wild type (“Zhongshu 6”) and transgenic (CM18-01, 03 and 04) ripe fruits. Values are mean ± standard error of three replicates. Significant differences are indicated with asterisks above the bars: *P ≤ 0.05, **P ≤ 0.01.

C5 and C6 volatile compounds in tomato, such as n-hexanal, (Z)-3-hexenal, (E)-2-hexanal, (Z)-3-hexenol, 1-pente-3-ol, 1-pente-3-one, and pentanal which are derived from fatty acids, are known to be formed by 13-TomloxC-HPL pathway^30, 31^. In order to further identify the role of *CmLOX18* in the C5 and C6 volatiles formation, GC-MS was used, and we detected a total of 26 volatile compounds from ripening fruit of wild-type and transgenic tomato at the breaker and B + 7 stages. Furthermore, the contents of C6 volatiles, containing hexanal, (Z)-3-hexenaland (Z)-3-hexen-1-ol were higher in transgenic fruits than that in wild-type fruits at the breaker and B + 7 stages. While the contents of C5 volatiles, including 1-pentanol and 1-pente-3-one, showed similar levels in transgenic and wild-type tomato (Fig. 8 and Table S2).

## Discussion and Conclusions

On the basis of the position of oxygenation sites in polyunsaturated fatty acids, plant LOXs can be classified as 9- and 13-LOXs. The positional specificity of plant LOXs is dependent on certain conserved sites at which the LOXs containing R/H, R/TF, R/S or R/CF, and R/TV motifs were predicted to be 13-LOX and 9-LOX, respectively^11^. However, some LOX proteins do not fit the predictive position-specificity models, and can produce both 9- and 13-hydroperoxy products^14, 17, 39^. In terms of the stereo-specificity of LOXs, the conserved single residue that is important for stereo control is an Ala for S-LOX and a Gly for R-LOX^40^. In our previous study, phylogenetic analysis grouped *CmLOX18* with the characterized members of 13-LOX, which contains an R/CF motif, and was predicted to be a 13S-lipoxygenase^37^. In the present study, the only detected reaction product of CmLOX18 was a 13-hydroperoxide, which was predominantly in the S configuration, as determined by HPLC analysis. Furthermore, kinetic studies showed that linoleic acid was the preferred substrate for recombinant *CmLOX18*, which maintained an intact LOX-like activity after being expressed in yeast, and was optimally active at pH 4.5. Similar to *CmLOX18*, pea *PsLOXN2*, which was involved in the late mechanisms of host resistance, exhibited normal enzyme activity at pH 4.5^41^. In addition, tea *CsLOX1*, which was involved in flower development and played a regulatory role in flower senescence, had a lower optimal pH of 3.6^17^. The acidic LOXs from rose and carnation might play important roles in flower senescence via membrane disruption by HPO and free radical action^42, 43^. In our previous studies, the expression level of *CmLOX18* was markedly increased when fruit developed to the climacteric stage, and was up-regulated by ethylene treatment^44^. These results indicated that the acidic *CmLOX18* might be involved in fruit ripening and play a regulatory role in the late development of melon.

Differential LOX functions arise from distinct localizations^26^. The subcellular location of LOXs might determine when and how precursors were fed into the allene oxide synthase (AOS) or hydroperoxide lyase (HPL) branches of the LOX pathway, followed by the synthesis of products with distinct biological properties^27^. The possible shift in the allocation of LOX-derived substrates to the HPL and AOS pathways and the substantial overlap of green leaf volatile (GLV)- and JA-regulated plant defence responses demonstrated a crosstalk between the two cascades that clearly exceeded the competition for a common substrate: the 13-hydroperoxide of linolenic acid^45–47^. In *Arabidopsis thaliana*, tobacco, and potato, there was co-localization of JA- and HPL-associated enzymes in the chloroplast^33, 48^. However, in this study, GLV-production *CmLOX18* was located in non-chloroplast organelles (Fig. 4), which was not consistent with the previous study. Similar results were also obtained for maize *ZmLOX10* ^26^. *ZmLOX8* ^49^ and *ZmLOX10*, which were involved in the AOS and HPL pathways, were located in chloroplasts and non-chloroplast organelles, respectively^26^. The discrepancy between our findings and those of previous studies could be explained by the fact that the GLV and JA pathways in melon and maize were physically separated from each other.

As has been mentioned previously, 13-lipoxygenases was key enzymes in the production of C5 and C6 compounds, and also JA^24^. For example, grape *VvLOXA* could be responsible for the formation of C6 compounds under acidic conditions^50^, whereas in olive, *Oep2LOX2* played a role in the biosynthesis of C6 and C5 volatile compounds^51^. The barley lox2:Hv:3 gene has been proposed to be involved in the channelling of linolenic acid substrate into the HPL-mediated production of C6 volatiles^52, 53^. In maize, *ZmLOX10* and *ZmLOX08* were specialized in providing substrates for the GLV and JA biosynthesis pathways, respectively^26^. In oriental melon fruit, straight-chain esters, such as ethyl acetate, have been demonstrated that they were the most important aroma components and might be derived from the fatty acid LOX–HPL metabolic pathways^38, 54^. Previous research has indicated that there are at least 18 LOX genes (*CmLOX01*–*CmLOX18*) in the melon genome^37^; however, the roles of these genes in the generation of fruit flavour volatiles have yet to be clarified. *CmLOX18* may, however, be an important candidate gene involved in straight-chain ester production in melon^37, 38^. In the present study, we transferred the melon *CmLOX18* gene into the tomato genome, which showed that *CmLOX18* might plays an important role in the synthesis of C6 compounds in melon fruits.

Previous research has indicated that the 13-LOX TomloxC is essential for the synthesis of C6 compounds^30, 31^. TomloxF, showing 13-LOX activity and preferentially useing linolenate as a substrate, was probably involved in the production of C6 volatile compounds^55^. In the present study, the contents of C6 volatile compounds, including hexanal, (Z)-3-hexenal, and (Z)-3-hexen-1-ol, in the fruits of transgenic tomato plants over-expressing *CmLOX18* were higher than those in wild-type fruits (Fig. 8 and Table S2). In addition, the over-expression of *CmLOX18* caused a significantly higher expression of *LeHPL* mRNA, whereas there was no change in the expression levels of *TomloxA*, *TomloxB*, *TomloxC*, *TomloxD*, *TomloxE*, and *TomloxF* mRNA (Fig. 7). These results indicated that the increases in C6 volatiles in transgenic fruits were attributable to the involvement of melon *CmLOX18* and tomato *LeHPL*, and were not related to endogenous tomato LOX. Furthermore, C6 volatiles and C5 compounds might be generated through an additional branch of the LOX pathway. Previous studies have shown that tomato *TomloxC* and olive *Oep2LOX2* are involved in the biosynthesis of C5 compounds^31, 41, 56^. However, in the present study, we could detect no significant differences between the C5 compounds, including 1-pentanol and 1-penten-3-one, of wild-type and transgenic fruits (Fig. 8). On the basis of the aforementioned results, we suspected that *CmLOX18* might be involved in the synthesis of C6 volatiles via its participation in the LOX–13HPL pathway, whereas it was not involved in the synthesis of C5 compounds. Moreover, *CmLOX18* and *TomloxD* shared 77% identity at the amino acid level, whereas the predicted amino acid sequence of *CmLOX18* displayed only 41–45% identity to other identified tomato LOXs. In addition to the high similarity in their amino acid sequences, both *CmLOX18* and TomloxD might also be involved in JA biosynthesis, given that previous studies have shown that *TomloxD* is involved in wound-induced JA biosynthesis and plant immunity to insect herbivores^27, 57^, and that the expression of *CmLOX18* mRNA is induced by wounding and methyl jasmonate treatments^58^.

In conclusion, the purified *CmLOX18* recombinant protein over-expressed in yeast showed positional specificity, as evidenced by its release of only a C-13 oxidized product. Linoleic acid was demonstrated to be the preferred substrate for the recombinant *CmLOX18* protein, which exhibited optimal catalytic activity at pH 4.5 and 30 °C. *CmLOX18*, which was localized to non-chloroplast organelles, played an essential role in the synthesis of C6 flavour volatiles in ripe fruits, but was not implicated in the synthesis of C5 compounds. Therefore, *CmLOX18* can be assumed to play an important role in C6 compound synthesis in melon fruits and might also be involved in JA biosynthesis in response to abiotic and biotic stresses.

## Materials and Methods

### Plant materials

Tomato *Solanum lycopersicum* L. cv. “Zhongshu 6” (WT) and the T_0_ and T_1_ transgenic tomato cultivars 35 S:: *CmLOX18* were individually planted in pots (volume of 25 L, soil: peat: compost = 1:1:1) in a greenhouse (Shenyang Agricultural University, Shenyang, China) in spring and fall seasons, 2015 to obtain the fruits for analysis of volatile compounds. Flowers were hand pollinated and tagged on the day of bloom for each plant. Fruit from WT and the transgenic tomato 45 day after pollination (DAP) were equivalent in age to breaker fruit. The harvested flesh tissue was froze immediately in liquid nitrogen and then stored at −80 °C until use.

### Heterologous expression and purification of Cm*LOX18* in yeast

Following the instructions provided by the manufacturer (Invitrogen, Paisely, UK), the *CmLOX18* cDNAs were cloned in the pYES2.1 TOPO-TA vector for regulated protein expression in yeast. Furthermore, all the constructs were transformed into the *Saccharomyces cerevisiae* cell line INVSc1 for expression of recombinant protein. Time-course studies *CmLOX18* gene expression in yeast was performed by harvesting an aliquot of cells at 0, 4, 8, and 24 hours after gala ctose induction.

The purification of recombinant LOX18 was performed according to the method described by Manriquez^59^.Protein purity was separated via a 12% Tris-glycine Coomassie brilliant blue stained SDS/PAGE.

### Western blot analysis

Western blot analysis was performed to detect the recombinant *CmLOX18* in yeast according to the method described by Patel *et al*.^60^. Total plant protein from ground leaves of transgenic tomato was extracted, quantified, separated and detected, by the method described by Mou *et al*.^61^ and NBT/BCIP buffer (Cat#00-2209, Zymed Lab., USA) system according to its manufacturer’s procedure.

### Characterization of the recombinant Cm*LOX18*

For pH optimum determination, glycin-HCl (pH 3.0, 3.5), CH_3_COOH-CH_3_COONa (pH 4.0, 4.5, 5.0, 5.5), Na_2_HPO_4_- Na_2_H_2_PO_4_ (pH 6.0, 6.5, 7.0, 7.5), and Tris-HCl (pH 8.0, 9.0) were used. LOX activities were determined according to the method described by Li *et al*.^54^. For the optimum temperature determination, 50 mM buffer was used for temperature range 20–45 °C increments of 5 °C. Each sample had three replicates. The kinetic parameters had been determined from a Michaelis-Menton plot in a range of substrate concentrations (linoleic acid and linolenic acid) between 15 and 350 μM.

According to the previous method^17^, we analyzed the *CmLOX18* products. The reaction mixture of enzyme activity was stopped by adding 0.1 M HCl solution, n-hexane was then added to the mixture. The 9-HPOD and 13-HPOD isomers were analysed by a SP-HPLC using a Slica Ultrasphere column (Inertsil® SIL-100A, 250 × 4.6 mm, 5 μm particle size) with a solvent system of n-hexane/2-propanol/acetic acid (100:2:0.1, by volume) and a flow rate of 1.0 ml min^−1^. The enantiomer composition was analyzed by chiral phase HPLC (CP-HPLC) on a Chiralcel OB-H column (Diacel Chem. Industries, 4.6 × 250 mm, 5 μm particle size) with a solvent system of n-hexane/2-propanol/acetic acid (100:5:0.1, by volume) and a flow rate of 1.0 ml min^−1^. The absorbance at 234 nm (conjugated diene system of the hydroxy fatty acids) was recorded. Standards of 9- and 13-HPOD were purchased from Larodan (Malm, Sweden).

### Subcellular localization analysis by transient expression of CmLOX18 in Arabidopsis protoplasts

Transient expression of GFP-tagged proteins *CmLOX18* cDNAs was isolated and cloned into the vector pBI221-GFP to get the C termini of the corresponding coding regions fused in-frame to the amino terminal end of GFP. GFP-tagged proteins were expressed in A. thaliana cells using polyethylene glycol (PEG) (Bio-Rad) approach for transient transformation^62^. Subcellular localization analysis was performed according to the method described by Cao *et al*.^65^.

### 35::CmLOX18 vector construction and tomato transformation

The full-length cDNA of *CmLOX18* was cloned into the Gateway-compatible vector p ENTR D-TOPO (Invitrogen). The amplicon was transferred by LR recombination to the binary vector pB7FWG2.0, which harbours two 35 S *Cauliflower mosaic vitus* (CaMV) promoters, the marker gene for Spectinomycin (Spe), bialaphos acetyltransferase genes (Bar), and enhanced green fluorescent protein (Egfp) (Fig. S1), following the manufacturer’s instruction. According to the previous method^63^, binary vectors containing the expected insert were subsequently transformed into Agrobacterium tumefaciens EHA105 cells by electroporation.

### PCR and Southern blotting

For PCR analysis, genomic DNA was extracted from transgenic lines and WT according to the manufacturer’s protocol (Tiangen Biotech Co.Ltd, Beijing, China). Froward primer TGTAGTGGTTGACGAT at + 768 and reverse primer TTGGAACTGACAGAAC at + 1085 of bar gene were used. For southern analysis, total DNA digested with HindШ restriction endonuclease, and electrophoresed in 0.8% agarose gels. Gels were transferred to nylon membranes and hybridized at 52 °C with DNA fragments labeled with a DIG High Prime DNA labeling kit (Roche, Switzerland). The blots were washed twice for 5 min at 65 °C with 40 min. NBT/BCIP was used for color detection.

### RNA Isolation and Real-time RT-PCR analysis

Total RNA from leaves and fruit samples were performed according to the manufacturer’s protocol (Tiangen Biotech Co.Ltd, Beijing, China) as describe previously^57^. For measuring the mRNA level of *CmLOX18*, *TomloxA-F*, and *LeHPL* in tomato fruits, the gene-specific primers of Real-time quantitative PCR (qPCR) were listed in the Table S1. The reaction of quantitative PCR was performed for gene expression studies according to the method described by Tang *et al*.^38^.

### Extraction and determination of volatiles

The volatiles of transgenic tomato fruit were exacted and detected according to the method described by Lewinsohn *et al*.^64^ and Tang *et al*.^38^ with a few modifications, in which an internal standard, namely 2-Octanone, (50 μL of, 59.5 mg/L, 0.5%, v/v, Aladdin Chemistry,China) was added to 10 mL supernatant of the juice for the determination of volatile matter content.

### Statistical and image analysis

The data were analyzed by the analysis of variance (ANOVA) using the SPSS13.0 statistics program, and statistical significance of differences were calculated by a one-way ANOVA following Duncan’s multiple range tests for each experiment at a P < 0.05 level. Origin (version 8.0) was used to chart. Plants were sprayed with Egfp and kept in the dark for a few minutes prior to imaging. NightSHADE LB 985 (Berthold, BadWildbad, Germany) was used to image enhanced green fluorescent protein.

## Electronic supplementary material

Figure S1;Figure S2;Table S1;Table S2
