# Supplementary material for: Melon13-lipoxygenase CmLOX18 may be involved in C6 volatiles biosynthesis in fruit
Source: Sci Rep. 2017 Jun 6;7:2816. doi: 10.1038/s41598-017-02559-6 (PMC5460189; doi:10.1038/s41598-017-02559-6)
Supplement: Supplementary file 1 — Figure S1;Figure S2;Table S1;Table S2 [file 41598_2017_2559_MOESM1_ESM.pdf]

Melon13-lipoxygenase *CmLOX18* may be involved in C6 volatiles biosynthesis in fruit

Chong Zhang[1 2†], Song-xiao Cao[1†], Ya-zhong Jin[1 3†], Li-jun Ju[1], Qiang Chen[1 4], Qiao-juan Xing[1], Hong-yan Qi [1*]

[1]Key Laboratory of Protected Horticulture of Education Ministry and Liaoning Province, College of Horticulture, Shenyang Agricultural University, Collaborative Innovation Center of Protected Vegetable Suround Bohai Gulf region, Shenyang, Liaoning 110866, PR China

[2]College of Horticulture, Jilin Agricultural University, Changchun, PR China

[3]College of Agriculture, Heilongjiang Bayi Agricultural University, Daqing, Heilong jiang163319, PR China.

[4] Experimental Center, Shenyang Normal University, Shenyang, Liaoning 110034, PR China

*corresponding author：hyqiaaa@126.com

† These authors contributed equally to the work

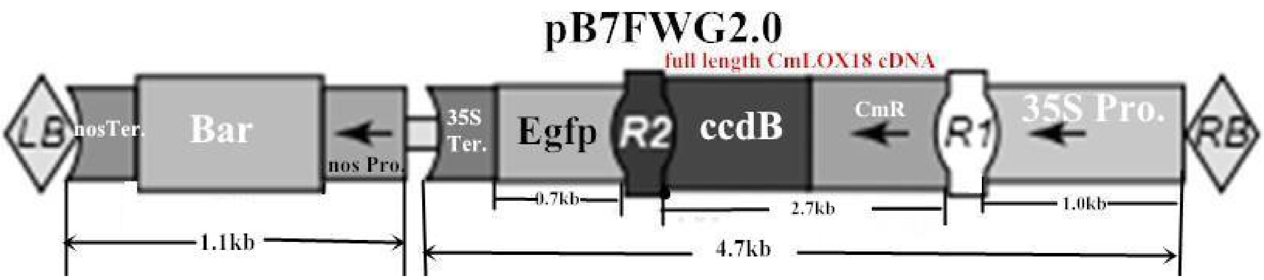

Figure S1 Schematic diagram of pB7WG2.0/LOX construct.

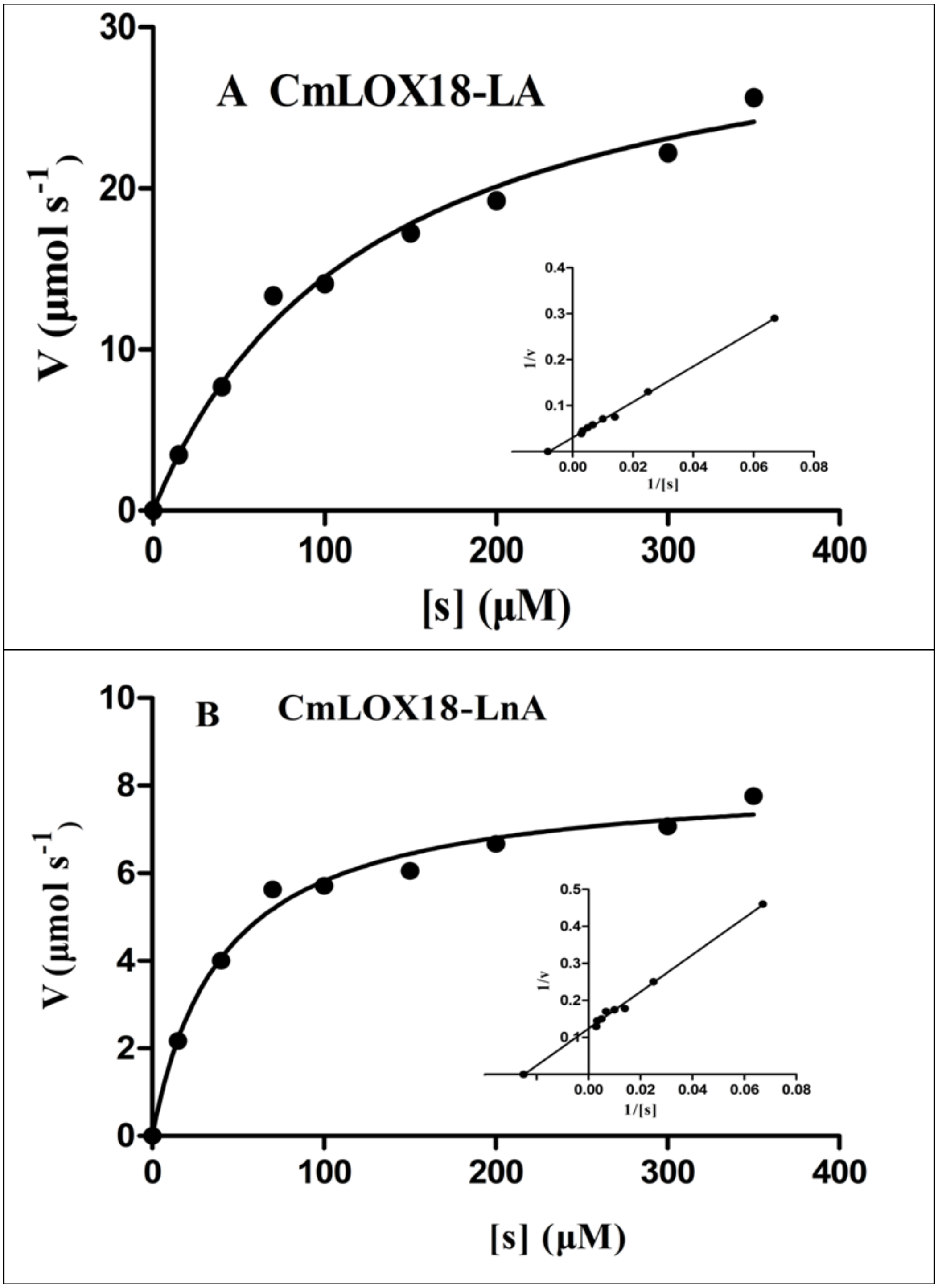

Figure S2 Effect of substrate concentration, linoleic acid (LA) and linolenic acid (LNA) on CmLOX18 enzymatic activity. Inserts are Lineweaver-Burk plots.

Table S1 Nucleotide sequences of primers used for PCR amplification in the present study.

Pimer name	Sequence (5'to 3')
CmLOX18-F	TGGAGACTATCACAATCG
CmLOX18-R	CTTTCCCATCACCTCTAA
LeHPL-F	AGCTACGGATTGCCGTTAGT
LeHPL-R	TCCACCACCATAATCC
TomloxA-F	TACCTCCCAAATCGTCCCAC
TomloxA -R	TGCGCTGTGATGGTTTTCAA
TomloxB-F	TGGCTTGGAAATTTGGGCAG
TomloxB-R	TCTTGTCGCCATGTCCTTCT
TomloxC-F	GCAATGCATCATGTGTGCTA
TomloxC-R	GTAAATGTCGAATTCCCTTCG
TomloxD-F	GGCTTGCTTTACTCCTGGTC
TomloxD-R	AAATCAAAGCGCCAGTTCTT
TomloxE-F	AAGCCGGAAATTCATGCCAG
TomloxE-R	TTCCAACCAATGTCTGCAGC
TomloxF-F	CCGAATCAAAGGGTGACTTT
TomloxF-R	GGTCTGTGATGATCGATTGC
Actin-F	TATGGTCAAGGCTGGGTTCG
Actin-R	CCATGCTCGATGGGGTACTT

Table S2 Overexpression CmLOX18 in fruits volatile compounds in wild type ("Zhongshu 6") and transgenic (CM18-01, 03 and 04) ripe fruits.

No	Aroma compounds	WT		CM18-01		CM18-03		CM18-04	
		Breaker	B+7	Breaker	B+7	Breaker	B+7	Breaker	B+7
	**Aldehydes(7)**								
1	Hexanal	76.6	62.3	108.5	99.7	133.1	112.2	122	100
2	cis-3-Hexenal	32.1	22.1	64.7	44.6	70.4	55.4	65.9	60
3	3-Furaldehyde	16.7	11.3	14.4	13.2	15.1	10.7	15.4	12.1
4	Heptanal	8.5	6.3	7.0	5.7	7.2	6.5	8.0	7.7
5	Benzaldehyde	nd	0.5	nd	0.4	nd	0.7	nd	0.6
6	phynylatablehyde	0.2	1.7	0.3	2.0	nd	1.5	0.5	1.9
7	Citralcistrans	nd	0.1	nd	nd	nd	0.2	nd	nd
	**Alcohols(6)**								
8	1-Pentanol	1.97	2.18	1.87	1.99	2.02	2.11	1.99	2.3
9	cis-3-Hexen-1-ol	15.7	19.7	30.7	38.9	33.8	45.5	31	47.2
10	5-methyl-5-Hexen-1-ol	nd	0.18	nd	0.1	0.5	0.35	nd	0.2
11	2-Heptyn-1-ol	nd	nd	nd	nd	1.7	1.7	nd	nd
12	Benezyl alcohol	0.12	0.88	nd	0.54	nd	0.81	nd	0.66
13	Phenethyl alcohol	nd	0.71	nd	0.74	nd	0.68	nd	0.69
	**Acids(5)**								
14	Acetic acid	4.12	1.35	4.02	1.37	3.92	1.29	4.3	1.4

15	Butanoic acid	0.2	nd	0.1	nd	0.1	nd	0.1	nd
16	Hexanoic acid	1.21	0.62	1.22	0.70	1.18	0.63	1.29	0.72
17	Octanoic acid	0.32	0.11	0.31	0.16	0.29	0.19	0.35	0.15
18	Pentadecanoic acid	0.1	nd	0.08	nd	0.09	nd	0.1	nd
	**Ketones(4)**								
19	1-Penten-3-one	nd	1.22	nd	1.44	nd	1.5	nd	0.9
20	6-Methyl-5-hepten-2-one	nd	0.09	nd	0.07	nd	nd	nd	0.07
21	Geranylacetone	nd	0.087	nd	0.09	nd	0.065	nd	0.076
22	beta-lonone	1.11	3.01	1.4	3.2	1.2	3.3	1.4	2.99
	**Others(4)**								
23	Birch-Me Methyl salicylate	nd	2.99	nd	3.1	nd	3.02	nd	3.0
24	Eugenol	1.73	4.89	1.55	5.3	1.65	5.1	1.75	7.3
25	2-Isobutylthiazole	0.16	nd	0.1	0.06	0.23	nd	0.17	0.09
26	Naphthalene	0.14	0.22	0.09	0.2	0.1	0.25	0.12	0.24
